# Stimulation of PGP Bacteria on the Development of Seeds, Plants and Cuttings of the Obligate Halophyte *Arthrocaulon (Arthrocnemum) macrostachyum* (Moric.) Piirainen & G. Kadereit

**DOI:** 10.3390/plants12071436

**Published:** 2023-03-24

**Authors:** José-María Barcia-Piedras, Jesús-Alberto Pérez-Romero, Enrique Mateos-Naranjo, Raquel Parra, Ignacio-David Rodríguez-Llorente, María Camacho, Susana Redondo-Gómez

**Affiliations:** 1 Centro Las Torres, Instituto de Investigación y Formación Agraria y Pesquera (IFAPA), Carretera, Sevilla-Cazalla de la Sierra Km 12.2, 41200 Alcalá del Río, Spain; mariag.camachomartinez@juntadeandalucia.es; 2 Departamento de Biología, Instituto Universitario de Investigación Marina (INMAR), Universidad de Cádiz, 11510 Puerto Real, Spain; 3 Departamento de Biología Vegetal y Ecología, Facultad de Biología, Universidad de Sevilla, Avda. Reina Mercedes s/n, 41012 Sevilla, Spain; 4 Departamento de Microbiología y Parasitología, Facultad de Farmacia, Universidad de Sevilla, 41012 Sevilla, Spain

**Keywords:** *Arthrocaulon (Arthrocnemum) macrostachyum*, halophyte, salinity, inoculant, PGP, cutting

## Abstract

The Earth is undergoing alterations at a high speed, which causes problems such as environmental pollution and difficulty in food production. This is where halophytes are interesting, due to their high potential in different fields, such as remediation of the environment and agriculture. For this reason, it is necessary to deepen the knowledge of the development of halophytes and how plant growth-promoting bacteria (PGP) can play a fundamental role in this process. Therefore, in this work were tested the effects of five PGP bacteria on its rhizosphere and other endophytic bacteria at different concentrations of NaCl on seed germination, plant growth (0 and 171 mM) and cutting growth (0 mM) of *Arthrocaulon macrostachyum*. The growth promotion in this strict halophyte is highlighted due to the presence of PGP bacteria and the fact that no salt is needed. Thus, without salt, the bacterial strains *Kocuria polaris* Hv16, *Pseudarthrobacter psychrotolerans* C58, and *Rahnella aceris* RTE9 enhanced the biomass production by more than 60% in both stems and roots. Furthermore, germination was encouraged by more than 30% in the presence of both *R. aceris* RTE9 and *K. polaris* Hv16 at 171 mM NaCl; the latter also had a biocontrol effect on the fungi that grew on the seeds. Additionally, for the first time in cuttings of this perennial species, the root biomass was improved thanks to the consortium of *K. polaris* Hv16 and *P. psychrotolerans* C58. Finally, this study demonstrates the potential of PGPs for optimising the development of halophytes, either for environmental or agronomic purposes.

## 1. Introduction

Halophytes are plants that are able to thrive in an environment with a concentration of NaCl greater than 200 mM [1]. In the past few decades, these plants have gained interest due to the potential that they present in different fields. In this way, thanks to their physiology, they can be used to desalinate soils degraded by excess salt [2,3], It has also been proven that they are effective phytoremediators against heavy metals [4]. In addition, halophytes are starting to be used as food for both livestock [5] and humans [6].

For these reasons, the ability of many halophytes to develop in both salt and salt-free conditions are well known [1,7,8,9]. Even multiplication by seeds is very well described for many of these halophilic species [10,11]. Despite this knowledge, no studies have been developed to optimise the propagation by asexual methods such as cuttings. This method of propagation is beneficial in the sense that plants are obtained faster than from seeds [12,13]. Furthermore, these plants are clones, thereby avoiding the variability that sexual reproduction introduces and could cause differences in the behaviour of the plant [14].

In addition, plants present a wide variety of relationships with other organisms. Among those interactions, those established with plant growth-promoting bacteria (or PGP bacteria) stand out from the rest due to their ability to enhance the plants’ conditions [15,16,17]. Specifically, there are many works that have used PGP bacteria to enhance the development of halophytes in different fields, such as for the restoration of saline ecosystems [18] or to increase the production of crops [19]. Among the bacterial properties that support plants are included those that provide nutrients, such as siderophore production for uptake iron [20] or phosphate solubilisation [21]. Other bacterial effects are related to the colonization of the root, e.g., acyl-homoserine lactone production [22] or the promotion of secondary roots via indole acetic acid production [23].

*Arthrocaulon macrostachyum* (Moric.) Piirainen & G. Kadereit was previously included in the genus *Arthrocnemum* as *Arthrocnemum macrostachyum* (Moric.) K. Koch, but the taxonomic nomenclature is a complex issue that is currently under evaluation [24,25,26]. This species is a perennial shoot succulent obligate halophyte that has been proposed to desalinate agricultural soils [27]; it is known that it is protected by its own endophytes at extreme concentrations of salt [28]. In this way, we hypothesised that PGP rhizobacteria isolated from different marsh plants could improve germination and growth, as well as improve the development of cuttings of *A. macrostachyum*. The propagation of *A. macrostachyum* has gained importance due to interest in the species as food [6,29,30] and for its nutritional and protective health benefits [31], in addition to its previously described bioremediation capacity.

Thus, in this work, there were three proposed objectives: (a) to study the effect that different bacteria with PGP properties (especially auxin-producing bacteria) have on plant growth and the physiology of *A. macrostachyum* grown both at 0 mM and at 171 mM NaCl; (b) analyse the effect of inoculation on germination of *A. macrostachyum* in non-saline and saline conditions; and (c) determine the rooting capacity of cuttings of *A. macrostachyum*.

## 2. Materials and Methods

### 2.1. Isolation of Bacteria

For bacterial isolates, soil samples of the rhizosphere of *A. macrostachyum* (5 cm deep) were collected from the Lebrija saltmarsh (36°54′ N–6°12′ W), characterised by having a loam-clay-sandy texture, a neutral pH, and an EC of 15 Sm^−1^. The isolates were obtained after mixing 1 g of soil in 99 mL of mineral sales [32], homogenising, and making serial decimal dilutions up to 10^−5^–10^−6^, which were spread on the surface in plates containing Plate Count Agar (PCA) (Difco^®^, Madrid, Spain) and 50% supplemented with cycloheximide (100 mg L^−1^, Sigma-Aldrich^®^, St. Louis, MO, USA) to prevent fungal growth. The same procedure was performed to add NaCl to the medium to a final concentration of 300 mM. The plates were incubated at 28 °C for 24–48 h until the appearance of individual colonies. After isolating colonies, the plates were incubated for an additional 72 h, and the slow-growing colonies were isolated. The purified strains were stored in cryogenic tubes with a mixture of peptone (0.5%) and glycerol (15%) at −80 °C. The strain RTE9, which was loaned from the collection of the Microbiology Department of the University of Seville, was isolated from the interior of an undetermined plant from the Rio Tinto saltmarsh. The origin of the bacterial strains used in the study is described in Table 1.

For the inoculation in the different tests, each frozen strain was spread on nutrient agar plates (NA) composed of nutrient broth (NB) (Scharlau^®^, Barcelona, Spain) with 16 g L^−1^ agar bacteriological (Difco^®^, Madrid, Spain) and grown at 28 °C for 48 h. For strains isolated in salt, NaCl was added to the culture medium to a final concentration of 300 mM. Next, a colony was taken and inoculated into tubes with 5 mL of NB that were incubated for 72 h at 28 °C with continuous agitation (180 rpm), obtaining a final bacteria concentration of 10^9^ mL^−1^.

For the inoculation of plant material (seed or plant), the bacterial cultures were centrifuged at 12,000 rpm for 5 min, and the cells were resuspended in 0.03 M magnesium sulphate buffer, until a bacteria concentration of 10^8^ mL^−1^ was achieved.

### 2.2. Molecular Characterisation of Bacteria: Amplification, Sequencing, and Phylogenetic Analysis of 16S rDNA

For the amplification of 16S rDNA, this work made use of the method described by Camacho et al. [33], in which 27F and 1492R primers were used [34]. The PCR products were purified with the Favorgen GEL/PCR Purification Kit (Biotech Corp^®^, Doral, FL, USA) and sequenced at the Sequencing Service of the University of Alcalá de Henares. To compare similarities with known sequences, the EzBioCloud database (https://www.ezbiocloud.net (accessed on 19 February 2023)) was used [35] (Table 1).

### 2.3. Bacterial Tolerance to High Salinity

For each strain, 10 μL of bacterial culture was placed on an NA plate with increasing concentrations of NaCl, between 0–25% (with 1% increments). Then, they were incubated for one week at 28 °C (*n* = 3).

### 2.4. Bacterial Tolerance to High Temperature

For each strain, 10 μL of bacterial culture was placed on an NA plate and incubated for two to seven days at 40 °C (*n* = 3).

### 2.5. Indole Acetic Acid Production (IAA)

To determine the IAA produced by the strains, the colorimetric method described by [36] was followed. The bacteria grew as described previously, adding 100 mg L^−1^ of L-Tyr to the NB. After growing, the cultures were centrifuged at 12,000 rpm for 5 min, and the supernatant was used to quantify the IAA concentration, adding the Salkowski reagent in a 1:4 (*v*:*v*). After 20 min at room temperature, the absorbance of the mixture was quantified (iMark Microplate Absorbance Reader, Bio-Rad^®^, Hercules, CA, USA) at 535 nm (*n* = 3), and the results were extrapolated with a standard line made from a stock of IAA (Panreac^®^, Barcelona, Spain) of 200 μg g^−1^ dissolved in 10% ethanol (serial dilutions between 0 and 200 μg g^−1^ with NB).

### 2.6. Acyl-Homoserine Lactone Production (AHL)

The bacteria grew as previously described and was centrifuged to determine AHL production. Then, 100 μL of supernatant was used for a colorimetric assay (*n* = 3) [37].

### 2.7. Siderophore Production

For each bacterium, 10 µL was added on a plate of CAS medium [38], which was incubated for 78 h at 28 °C. Subsequently, the production of siderophores was quantified by measuring the diameter of the hydrolytic halo formed (*n* = 3).

### 2.8. Phosphate Solubilisation

For phosphate solubilisation, 10 µL of bacterial cultures was placed in a plate of PVK medium [39] and kept at 28 °C for five days. Then, the activity was assessed according to the generated hydrolytic halo (*n* = 3).

### 2.9. Vegetal Material

In this research, commercial seeds of rapeseed (*Brassica napus* L.) were used. In addition, seeds of *A. macrostachyum* were recollected from the saltmarsh of the Odiel River, SW of the Iberian Peninsula (37°13′4.4″ N–6°57′35.3″ W) in mid-September of 2014 for experiments 1 and 2, and 2015 for experiment 3. Once the seeds were collected, they were kept at 5 °C until use. 

For the disinfection of seeds of *B. napus*, the protocol specified by [40] was followed, and for *A. macrostachyum*, the seeds were immersed 30 s in 96% H_2_SO_4_, followed by 7 washes with sterile water, sodium hypochlorite 5% for 3 min, then finally another 7 washes with sterile water.

### 2.10. Inoculation

A solution of MgSO_4_ 0.03 M was used as an inoculation control. For the seeds of *B. napus* and *A. macrostachyum*, the bacteria were cultivated as described previously (see the Indole Acetic Acid Production section). Then, the seeds of both species were inoculated as described by Patten and Glick [36].

In experiment 1, both seedlings and three-month-old plants of *A. macrostachyum* were inoculated with 200 μL and 1 mL of bacterial suspension (10^8^ cells ml^−1^), respectively.

For the cuttings of *A. macrostachyum* (experiment 3), they were immersed in 100 mL of bacterial suspension (10^8^ cell ml^−1^) for one hour immediately after they had been cut.

Throughout the experimental period, seedlings, plants, and cuttings from experiments 1 and 3 were reinoculated once per month (ensuring the presence of bacterial strain).

### 2.11. Rapeseed Root Elongation Test

**Figure 1:**
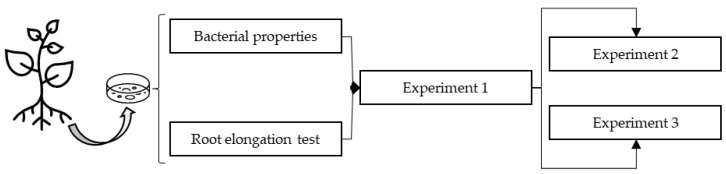
Organization chart of experimental design indicating the order and correlations between the tests and experiments carried out in this research.

To select the strains that were applied in experiment 1, a rapeseed test was previously performed (Figure 1). This species is sensitive to IAA changes, which are reflected in the elongation of roots [41]. Therefore, after inoculating 40 seeds of *B. napus* for each treatment, they were distributed into four sterile growth bags (DIK-710A, Daiki^®^, Kounosu, Japan) containing 12 mL Hewitt solution 0.5× [42]. The bags were placed in a growth chamber (Sanyo Electric^®^, Osaka, Japan) with a 12 h light and 12 h dark cycle, illuminated by a light source (λ = 400–700 nm) with a flux density of 250 μmol m^−2^ s^−1^, and 80% relative humidity for seven days. After this period, the length of the radicle was measured.

### 2.12. Experiment 1: Influence of Bacterial Strains and Salt on Plant of A. macrostachyum

The strains selected in the rapeseed root elongation test were used to inoculate the seeds of *A. macrostachyum* (Figure 1). One hundred inoculated seeds per treatment were placed in 4 Petri dishes with 1% agar-water. After this, the plates were kept in a growth chamber (Sanyo Electric^®^, Osaka, Japan) with a cycle of 10 h of light at 20 °C and 14 h of darkness at 5 °C for one month.

Then, the seedlings were transplanted into perforated seedbeds with a substrate composed of 2:1 perlite-vermiculite (*v*/*v*). In addition, the seedbeds were placed in trays that were filled with Hewitt 0.5× solution to a depth of 1 cm. Previously, all materials were sterilised by moist heat sterilisation (60 min at 121 °C). The seedbeds were placed in the greenhouse of the IFAPA Center Las Torres in Seville (37°30′42.4″ N–5°57′47.4″ W) with a cycle of 16 h of light at 30 °C and 8 h of darkness at 20 °C for three months. One day after transplanting, the seedlings were inoculated.

Next, the length, central diameter, and surface area of the main branch and the number of secondary branches were quantified (*n* = 20). Then, plants were placed in sterile Leonard jars with a 2:1 perlite–vermiculite (*v*/*v*) ratio. In order to determine the effect of the presence of salt in *A. macrostachyum* with respect to the inoculated strains, 10 plants used 0.2 L of Hewitt 0.5× nutrient solution as the liquid phase; in 10 other plants, the same nutrient solution was enriched with 171 mM NaCl (keeping both the volume and concentration of the nutrient solution constant). The plants grew for three months under identical environmental conditions. Reinoculation was performed one day after the transplant.

### 2.13. Experiment 2: Effects of Bacterial Inoculation and Salt on the Germination of A. macrostachyum

With the bacterial treatments from experiment 1 that promoted the most growth of *A. macrostachyum*, a new germination test was performed (Figure 1). This germination experiment was performed in the same manner as experiment 1, except for the number of seeds (fifty seeds were germinated without salt and another fifty were germinated with a salt concentration of 171 mM per treatment). The germinations were recorded daily to calculate the final germination percentage, number of days until the first and last germination, and mean time to germinate (MTG) [43]. Lower MTG values indicate faster germination. Furthermore, to determine whether the bacterial treatment could inhibit the growth of fungi associated with seeds, counts of the apparent colony forming units (CFUs) on the plates were made at the end of the experiment.

### 2.14. Experiment 3: Influence of Bacterial Inoculation on Cutting of A. macrostachyum

Cuttings of *A. macrostachyum* were obtained by sectioning the first 5 cm of the new tertiary branches from a 2-year-old plant grown in a greenhouse with Hoagland solution [44] 1% NaCl (Figure 1). In total, 200 cuttings were obtained (40 per treatment and 40 cuttings for the initial measurements). After inoculation, each cutting was placed 2 cm deep in a perforated seedbed filled with coconut fibre and perlite 5:1 (*v*/*v*). The seedbeds were placed in trays (*n* = 4) that were kept with a constant volume of Hoagland solution. The experiment was carried out in the greenhouse of the University of Seville (37°21′32.6″ N–5°59′13.4″ W) at 21–25 °C, a relative humidity of 40–60%, and 16 h of natural light (250 μmol m^−2^ s^−1^ minimum and 1000 μmol m^−2^ s^−1^ maximum) for 60 days.

### 2.15. Gas Exchange

Experiment 1: Just before harvesting the plants, photosynthetic gas exchange was measured (*n* = 5) using an infrared gas analyser (IRGA) in an open system (Lci portable photosynthesis, ADC^®^, UK) [45]. The environmental parameters were: 30–35 °C, 45% relative humidity, atmospheric CO_2_ concentration 400 μmol mol^−1^, and photon flux density 1000 μmol m^−2^ s^−1^.

Experiment 3: Prior to harvest, gas exchange was measured in the same way as experiment 1, except that seven replicates per treatment were made with IRGA LI-6400 (LI-COR^®^, Lincoln, NE, USA). Additionally, the photosynthetic light had a 15% blue light (to maximise stomatal aperture), a vapour pressure deficit of 1.5 ± 0.1 kPa, and an air temperature of 24 ± 0.5 °C.

In both experiments, due to the branch of *A. macrostachyum* being cylindrical, the photosynthetic surface was considered to be half of the total photosynthetic surface of the branch and the photosynthetic parameters were calculated as described [45].

### 2.16. Plant Growth

Experiment 1: At the end of the experimental period, the plants were harvested, and the aerial parts and root were weighed (fresh weight). Subsequently, they were dried in an oven (at 60 °C for 48 h), and they were weighed again (dry weight) to calculate the water content.

Experiment 3: At the beginning of the assay, measurements of the length and diameter of all the cuttings were taken. Forty of them were dried and weighed (initial dry weight) as described in experiment 1. After two months, the number of alive cuttings, length and diameter of branches, and the fresh weight of both branches and roots were measured. Then, the cuttings were placed in plastic slider zip bags with distilled water at 5 °C in the dark. After 24 h, the surfaces of the plants were dried and weighed to determine the turgid weight. Finally, they were dried to confirm the dry weight of both the branches and the roots. With these data, the relative water content (RWC) [46] and the relative growth rate (RGR) [47] were calculated.

### 2.17. Statistics

Statistical analyses were developed using SPSS 21. The results correspond to the mean ± standard error (S.E.). For continuous variables, normality was tested with the Kolmogorov–Smirnov test and the Brown–Forsythe test to verify homoscedasticity. In order to test for differences, the Kruskal–Wallis test was used for the discrete variables, and an ANOVA was used for the continuous variables. For post-hoc tests, the Mann–Whitney U and Fisher’s LSD test were used, both with a significance threshold of *p* < 0.05.

## 3. Results

### 3.1. Bacterial Properties

Regarding the physical properties, all of the strains grew in at least 1% NaCl, and the strain that withstood the highest salinity was Hv16 at 9% NaCl. Only Hvs13 had sufficient tolerance to grow at 40 °C (Table 2).

In relation to PGP properties, the only property present in all strains was the production of IAA, with Hv16 producing the highest amount (Table 2). AHL production was only present in strains Hv16 and Hvs76, the former being the largest producer of AHL (Table 2). The siderophore production occurred in all strains except C59 and RTE9, and Hv16 repeated producing more siderophores than any other strain (Table 2). Finally, the phosphate solubilization was only present in RTE9 (Table 2).

### 3.2. Rapeseed Root Elongation Test

**Figure 2:**
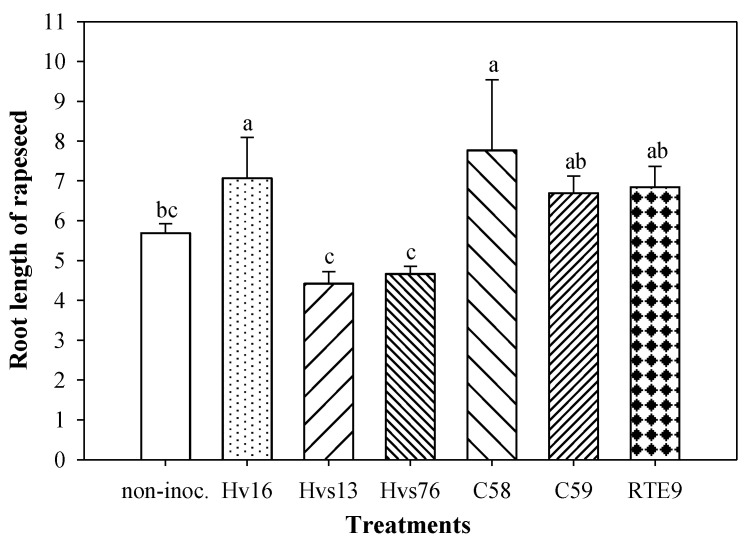
Radicle length in rapeseed seedlings (*Brassica napus* L.) inoculated with six different bacteria, after 7 days. Values correspond to the mean ± S.E. (*n* = 4). Different letters indicate that the mean values are significantly different from each other (Fisher’s LSD test, *p* < 0.05).

The major root elongation values obtained for the treatments inoculated with C58 and Hv16 and were found to be approximately 30% longer than the non-inoculated control (Fisher’s LSD test, *p* < 0.05). In addition, the treatments C59 and RTE9 were also selected, which presented a greater root length than the control (14 and 19%, respectively), although they did not show significant statistical effects (Figure 2).

### 3.3. Influence of Bacterial Strains and Salt on Plant of A. macrostachyum

#### 3.3.1. Plant Growth

After the first three months of growth of the seedlings of *A. macrostachyum*, it was observed that the branch length of the treatments Hv16 and RTE9 were 32% and 23% longer than the control, respectively. A similar trend was observed on the surface area. Furthermore, regarding the number of secondary branches, the Hv16 treatment accelerated the branching of the seedlings. However, no significant difference was observed for the diameters (Table 3). At the end of experiment 1, strains Hv16, C58, and RTE9 promoted an increase in both above- and below-ground biomasses of more than 60% compared to the non-inoculated treatment (Fisher’s LSD test; *p* < 0.05) in the absence of salt (Table 4). In the treatments at 171 mM NaCl, growth stimulation was observed exclusively at the radical level, with the Hv16, C59, and RTE9 treatments presenting at least 50% more biomass than the non-inoculated treatment (Fisher’s LSD test, *p* < 0.05) (Table 4). On the other hand, no treatment increased the water content of the plants.

#### 3.3.2. Gas Exchange

At a concentration of 0 mM, all inoculated treatments showed higher values than the control in g_s_, and the Hv16 and RTE9 treatments also presented higher values in A and C_i_ (Fisher’s LSD test, *p* < 0.05). Conversely, there were no differences at 171 mM NaCl (Fisher’s LSD test, *p* < 0.05). Regarding the iWUE, only the RTE9 171 mM treatment had an efficiency greater than 60% in comparison to the non-inoculated treatment (Fisher’s LSD test, *p* < 0.05) (Table 5).

### 3.4. Effects of Bacterial Inoculation and Salt on the Germination of A. macrostachyum

**Figure 3:**
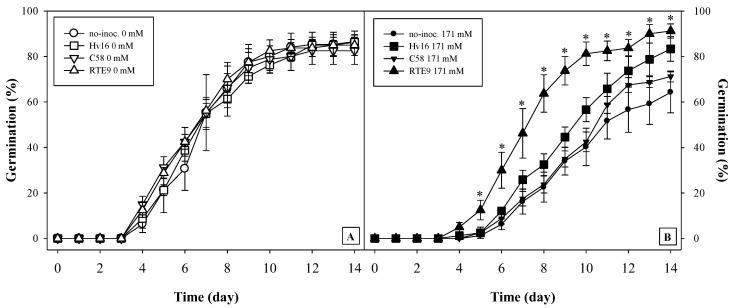
Cumulative germination percentage of *A. macrostachyum* grown at 0 (**A**) and 171 (**B**) mM NaCl non-inoculated (ο), or inoculated with Hv16 (☐), C58 (∇) and RTE9 (Δ) bacterial strains over 14 days. Values correspond to the mean ± S.E. (*n* = 4). Asterisks indicate that there were significant differences between mean values are for that day (Mann–Whitney U test; *p* < 0.05). Treatments 0 and 171 mM were analysed independently.

In the treatments without salt, no germination parameters were affected by inoculation (Figure 3A and Table 6) (Fisher’s LSD test, *p* < 0.05). On the contrary, in presence of salt, the non-inoculated treatment had a deceleration in the curve of germinated seeds from the fifth day (Table 6). Meanwhile, the Hv16 and RTE9 treatments did not show that delay (Figure 3A) and, furthermore, reached the highest percentages of germinated seeds (Table 6).

With regard to the biological control of fungi, more CFUs appeared in the saline treatments (Table 6). The biocontrol of the Hv16 strain at 171 mM was remarkable, as it reduced the presence of CFUs by 50% as compared to the non-inoculated treatment (Table 6).

### 3.5. Influence of Bacterial Inoculation on Cutting of A. macrostachyum

#### 3.5.1. Plant Growth 

No significant differences were observed in the branches of cuttings, although a trend was observed in which the inoculated cuttings were slightly longer than the non-inoculated cuttings (Table 7). On the other hand, focusing on radical growth, it was observed that strain Hv16 promoted 20% more radical elongation than the control treatment. Furthermore, this treatment together with C58 stimulated the production of roots, so that the dry weight of both was about 20% higher than that of the treatment without inoculation (Table 7).

#### 3.5.2. Gas Exchange

Regarding the photosynthetic parameters, only the net photosynthesis rate and the _i_WUE presented statistical differences (Fisher’s LSD test, *p* < 0.05). Thus, for parameter A, the treatments with the highest rate were RTE9 and C58, reaching values that were 28% and 14% higher than the control treatment, respectively (Table 8). Regarding the _i_WUE parameter, the RTE9 strain doubled the value of the efficiency with respect to the non-inoculated cuttings, and the Hv16 strain was 36% higher than the control (Table 8).

## 4. Discussion

The marshes of the south of the Iberian Peninsula are characterised by extreme conditions such as high temperatures and salinities. Under the same conditions, the bacteria used in this research were isolated, which strongly concur with the first citation for each bacterial species. Thus, *K. polaris* came from desert areas [48], and *A. psyichrotolerans* was also isolated from an extreme environment, but in this case, a dry and cold environment [49]. Finally, other species appear in an extreme environment, not due to atmospheric conditions but due to the presence of a compound in high concentrations. Accordingly, *Ensifer meliloti* is a rhizobia isolated from high-salinity areas [50], and *Brevibacterium frigoritolerans* was described in soil with high concentration of lead [51]. In addition to the ability to live in extreme environments, all bacteria have been described as PGP bacteria (either by their properties or by testing on any plant); it is important to note that *K. polaris* was never described as such until this investigation.

Considering the germination of the seed, many species that are halophilic at maturity are sensitive to salinity at germination [52,53,54]; specifically, *A. macrostachyum* has been described as such, as salinity affects the germination process [11,55,56], although if the seed is recovered in a non-saline medium after the exposure to salinity, the germination percentage will be higher than treatment without salt [57,58,59]. In this way, the bacteria used had a fundamental role when the plant is exposed to NaCl in germination. Several aspects could be highlighted in this phase; the most important could be the way in which the presence of the strains Hv16 (*K. polaris*) and RTE9 (*R. aceris*) allow germination percentages similar to those detected when there is no salt stress to be obtained. Although there is no work in which a halophyte was inoculated with any strain of *R. aceris*, Vyas et al. used a strain of *Rahnella* sp., isolated from a halotolerant species such as buckthorn, for inoculating barley, chickpea, pea, and maize seeds and observed that germination of all of them was improved under the controlled saline environment [60]. Furthermore, in the 171 mM NaCl treatment with the RTE9 strain, an acceleration in germination was observed (the slope of its curve is more pronounced, Figure 3B). This process indicates that, at a certain point in time, there were always more germinated seeds if they were inoculated with RTE9. That is, from an ecological point of view, more individuals are recruited in less time, which is an advantage against possible factors that hinder the development of new individuals of *A. macrostachyum* (predation, flood, temperature, salinity, etc.). Considering the final germination percentage of this work, the values were similar to those described by [28], who inoculated *A. macrostachyum* seeds with endophytic bacteria of the same species at 154 mM NaCl; however, in this work, we also obtained an acceleration in seedling recruitment. The explanation for this improvement in germination can be attributed to the presence of IAA produced by bacteria, because this hormone influences some enzymes such as α-amylase that facilitate the assimilation of starch, which in turn induces early germination, even in stressful situations [61]. Another remarkable aspect is the biocontrol of the fungi associated with the seed, which are difficult to eradicate, despite having treated the seeds with sulphuric acid. In this sense, the Hv16 strain was shown to control the proliferation of fungi in vitro and in the presence of salt, and although this species has not been described as a biocontroller, other species of its genus (*Kocuria rosea*) have been described as such in tubers of potato [62].

In this work, the development of *A. macrostachyum* has been favoured through inoculation with PGP bacteria and without the need for NaCl, despite being an obligate halophyte [45,63]. Thus, in the first months after germination, an increase in the photosynthetic surface of *A. macrostachyum* grown in the absence of salt was obtained (stressful environment for an obligate halophyte). This fact is reflected both by increasing the length of the main branch and the presence of more secondary branches. This last effect coincides with the data obtained by [64] in the halophyte *Salicornia bigelovii* when inoculated with strains, individually or in consortium, isolated from plant rhizospheres of the same ecosystem. In the next phase of experiment 1, differences were also observed in the effect that strain has on plant growth, depending on the salinity of the medium. Thus, when *A. macrostachyum* grows without salt, inoculation with PGP bacteria led to greater biomass, both in branches and roots. This improves the results obtained by [28], who did not detect changes in these same parameters in *A. macrostachyum* inoculated with endophytes under the same conditions. Furthermore, for almost all the inoculated treatments with NaCl, a benefit was detected in the roots, for there occurred at least a doubling the underground biomass. These results represent an advancement in the field of increasing the biomass of halophyte roots by PGP bacteria, because previous works using *Salicornia europaea* inoculated with a nitrogen-fixing PGP bacterium [65] or *A. macrostachyum* inoculated with PGP endophytes [28] did not detect such improvement in the roots.

In the presence of salt, no effect of inoculation was observed in *A. macrostachyum*; this observation could be because the inoculum promotes plant growth, especially in the presence of factors that are stressful for the plant [66,67,68]. However, in the case of a strict halophyte, it seems that the presence of an optimal concentration of NaCl stimulates the growth of *A. macrostachyum,* hiding the positive effect that was observed when the plant grows under stressful conditions (without salt). Furthermore, the effectiveness of PGP bacteria to depend on the environment in which they are found has already been described; that is, the saline environment could affect the expression of the PGP properties of bacteria [69]. In this way, *P. psychrotolerans* decreased IAA production in wheat crops planted in soil contaminated with NaCl [70].

Regarding gas exchange, a similar trend was observed, although less pronounced, than that described for biomass. Thus, in a medium without salt, unfavourable for *A. macrostachyum*, the highest photosynthetic rate was obtained in the inoculated treatments in which an increase in biomass was also described. This fact is an advance over previous studies with this species in salt-free conditions and inoculated with other bacterial strains [28]. In contrast, the treatments containing salt did not show photosynthetic differences (or were slightly lower in C_i_) compared to the non-inoculated treatment with inoculated treatments. In part, the homogeneity in gas exchange could be due to the fact that the photosynthetic apparatus of halophytes is adapted to salinity, as described by Lu et al. [71] in the halophyte *Suaeda salsa*. Although this difference could be compensated by the effect that salt has on the photosynthetic surface of *A. macrostachyum*, because this species between 171 and 510 mM NaCl increases the photosynthetic surface, thanks to an increase in the diameter of its branches [45]. In other words, the treatments of 171 mM NaCl balance the lowest photosynthetic rate with a larger photosynthetic surface (because they present a greater amount of branch biomass). Furthermore, as previously mentioned, the presence of an optimal concentration of salt could be hiding the effect of the bacterial strains on the aerial biomass.

Finally, the rooting of cuttings is a process to consider because it allows quickening the production of plants, especially of perennial species, in which producing plants from seeds is a slower process. In addition, thanks to this process, clones are produced which can solve a specific need that is required from this plant. Specifically, *A. macrostachyum* has been described as a phytodesalination plant [27] and by cuttings, a large number of individuals with the most appropriate characteristics could be obtained to be able to phytoremediate a contaminated soil. In nature, many species of perennial shrubs reproduce agamically by rooting their lower stems. This portion can be separated from the plant and used for vegetative multiplication [72]. In fact, there exists research in which fragments of *A. macrostachyum* are used, such as that of Paraskevopoulou et al. [73], who described that cuttings of this species can be used for green roof systems in arid areas. In our case, we can add that the inoculation stimulated root growth of cuttings, which may be due to a higher production of auxins and higher values of A in all inoculated treatments. In fact, other authors have described positive results that agree with the values obtained in this work with respect to the rooting of cuttings assisted by IAA-producing bacteria at the greenhouse, in the way that was described for *Olea europaea* [40], and the manner that Zenginbal and Demir performed for two species of the genus *Morus* [74].

## 5. Conclusions

In this work, the most significant finding is that the strains *K. polaris* Hv16 and *P. psychrotolerans* C58 (PGP bacteria isolated from marshes) enhance the development of *A. macrostachyum* cuttings by stimulating root biomass. In relation to germination, the strains *R. aceris* RTE9 and *K. polaris* Hv16 compensate for the negative effect produced by NaCl on the seeds, increasing the germination percentages under these conditions; in addition, Hv16 also reduces the development of fungi in the seeds in the presence of salt. Finally, thanks to the inoculants Hv16, C58, and RTE 9, the development of the biomass of branches and roots is enhanced in the strict halophyte *A. macrostachyum* grown without salt; even in optimal salt conditions, there is a positive synergy between these strains and salt that induces greater root production in *A. macrostachyum*.

## Figures and Tables

**Table 1:** Strains and environments from which the bacteria have been isolated and closest species to bacteria used in this work based on 16S rRNA partial sequences.

Strain	Environment	Related Species	Identity (%)	Accession Number
Hv16	Rhizosphere of *A. macrostachyum* from Guadalquivir River saltmarsh.	*Kocuria polaris*	98.6	LN794848
Hvs13	Rhizosphere of *A. macrostachyum* from Guadalquivir River saltmarsh (isolated at 350 mM NaCl).	*Brevibacterium frigoritolerans*	100	OU596762
Hvs76	Rhizosphere of *A. macrostachyum* from Guadalquivir River saltmarsh (isolated at 350 mM NaCl).	*Pseudarthrobacter siccitolerans*	99.4	OU596760
C58	Rhizosphere of *A. macrostachyum* from an irrigation canal close to Guadalquivir River saltmarsh.	*Pseudarthrobacter psychrotolerans*	99.3	OU596763
C59	Rhizosphere of *A. macrostachyum* from an irrigation canal close to Guadalquivir River saltmarsh.	*Ensifer meliloti*	99.9	OU596759
RTE9	Interior of an indeterminate plant of the Tinto River saltmarsh.	*Rahnella aceris*	99.3	OU596761

**Table 2:** Quantification of the properties of the bacterial strains: salinity range (SAL), tolerance to grow at 40 °C (TEM), indole acetic acid production (IAA), relative acyl-homoserine lactone production (AHL), siderophore production (SID), and phosphate solubilization (PHO) (*n* = 3).

Strain	SAL (%)	TEM	IAA (μg g^−1^)	AHL (%)	SID (mm)	PHO (mm)
Hv16	0–9	−	200	50	20	−
Hvs13	0–6	+	50	75	10	−
Hvs76	0–4	−	150	100	10	−
C58	0–4	−	150	25	10	−
C59	0–1	−	100	50	-	−
RTE9	0–4	−	150	0	-	30

**Table 3:** Length, diameter, surface area of main branch, and number of secondary branches of seedlings of *A. macrostachyum* inoculated with the different bacteria after 90 days. Values correspond to mean ± S.E. (*n* = 20). Different letters indicate that the mean values are significantly different from each other (Fisher’s LSD test for all variables except number of secondary branches, for which Mann–Whitney U test was used, both *p* < 0.05).

Treatment	Branch Length (cm)	Branch Diameter (mm)	Branch Surface Area (cm^2^)	Secondary Branches
Non-inoculated	2.2 ± 0.2 ^b^	1.7 ± 0.1 ^ab^	1.2 ± 0.1 ^c^	0 ± 0 ^c^
Hv16	2.9 ± 0.2 ^a^	1.9 ± 0.1 ^a^	1.7 ± 0.1 ^a^	2 ± 0 ^a^
C58	2.1 ± 0.2 ^bc^	1.9 ± 0.1 ^a^	1.4 ± 0.1 ^ab^	1 ± 0 ^bc^
C59	2.0 ± 0.2 ^c^	1.6 ± 0.1 ^bc^	1.1 ± 0.1 ^c^	1 ± 0 ^bc^
RTE9	2.7 ± 0.2 ^a^	1.8 ± 0.1 ^a^	1.7 ± 0.2 ^a^	1 ± 0 ^bc^

**Table 4:** Dry weight of aerial part and roots and water content of aerial part of *A. macrostachyum* grown at 0 and 171 mM NaCl with and without inoculation with three different bacterial strains after 180 days. Values correspond to the mean ± S.E. (*n* = 10). Different letters indicate that the mean values are significantly different from each other (Fisher’s LSD test, *p* < 0.05). Treatments 0 and 171 mM are analysed independently.

	0 mM NaCl		
Treatment	Aerial Dried Weight (g)	Root Dried Weight (g)	Water Content (%)
Non-inoculated	0.27 ± 0.10 ^c^	0.19 ± 0.06 ^c^	84.1 ± 1.8 ^ab^
Hv16	0.59 ± 0.12 ^a^	0.73 ± 0.18 ^a^	85.3 ± 0. 8 ^a^
C58	0.45 ± 0.09 ^ab^	0.81 ± 0.23 ^a^	78.9 ± 1.2 ^c^
C59	0.34 ± 0.07 ^abc^	0.40 ± 0.11 ^bc^	80.4 ± 0.7 ^bc^
RTE9	0.57 ± 0.13 ^ab^	0.67 ± 0.19 ^ab^	81.4 ± 0.5 ^abc^
	**171 mM NaCl**		
Non-inoculated	1.25 ± 0.27 ^ab^	0.75 ± 0.18 ^c^	87.6 ± 0.4 ^ab^
Hv16	1.66 ± 0.30 ^a^	1.14 ± 0.15 ^ab^	88.0 ± 0.2 ^ab^
C58	0.89 ± 0.15 ^b^	0.77 ± 0.13 ^bc^	88.6 ± 0.9 ^ab^
C59	1.84 ± 0.32 ^a^	1.21 ± 0.19 ^ab^	87.4 ± 0.6 ^b^
RTE9	1.93 ± 0.23 ^a^	1.28 ± 0.13 ^a^	87.7 ± 0.5 ^ab^

**Table 5:** Net photosynthetic (A) and stomatal conductance (g_s_) rates, intercellular CO_2_ concentration (C_i_) and intrinsic water use efficiency (_i_WUE) of *A. macrostachyum* grown at 0 and 171 mM NaCl, with and without inoculation with four different bacterial strains after 180 days. Values correspond to the mean ± S.E. (*n* = 5). Different letters indicate that the mean values are significantly different from each other (Fisher’s LSD test, *p* < 0.05). Treatments 0 and 171 mM are analysed independently.

Treatment	0 mM NaCl			
	A (μmol m^−2^s^−1^)	g_s_ (mmol m^−2^s^−1^)	C_i_ (μmol mol^−1^)	_i_WUE (mmol mol^−1^)
No-inoculated	7.5 ± 1.4 ^c^	90 ± 20 ^c^	215 ± 20 ^bc^	85 ± 12
Hv16	14.1 ± 1.0 ^a^	240 ± 30 ^a^	250 ± 6 ^a^	70 ± 14
C58	8.6 ± 1.7 ^bc^	120 ± 40 ^b^	215 ± 17 ^bc^	82 ± 12
C59	5.8 ± 1.7 ^c^	90 ± 20 ^c^	224 ± 19 ^ab^	78 ± 12
RTE9	10.9 ± 1.6 ^ab^	150 ± 30 ^b^	250 ± 10 ^a^	76 ± 16
	**171 mM NaCl**			
No-inoculated	7.3 ± 0.7	140 ± 20	275 ± 7 ^a^	53 ± 4 ^b^
Hv16	5.8 ± 1.1	100 ± 30	252 ± 9 ^ab^	61 ± 7 ^ab^
C58	6.8 ± 1.2	90 ± 20	232 ± 19 ^ab^	86 ± 15 ^ab^
C59	5.5 ± 0.6	90 ± 10	246 ± 19 ^ab^	69 ± 13 ^ab^
RTE9	5.6 ± 0.9	90 ± 40	215 ± 13 ^b^	86 ± 1 ^a^

**Table 6:** Germination parameters and number of colonies forming units (CFU) of seeds of *A. macrostachyum* at 0 and 171 mM NaCl with and without inoculation with four different bacterial strains after 30 days. Values correspond to mean ± S.E. (*n* = 4). Different letters indicate that the mean values are significantly different from each other (Fisher’s LSD test for total germination and MTG, Mann–Whitney U test for first and last germination and number of fungal colonies, both *p* < 0.05). Treatments 0 and 171 mM are analysed independently.

	0 mM NaCl				
Treatment	Total Germination (%)	First Germination (d)	Last Germination (d)	MTG (d)	CFU
Non-inoculated	86 ± 5	5 ± 1	11 ± 1	7.2 ± 0.6	2 ± 0
Hv16	86 ± 3	4 ± 1	12 ± 1	7.2 ± 0.3	2 ± 0
C58	83 ± 6	4 ± 0	11 ± 2	6.6 ± 0.3	3 ± 1
RTE9	88 ± 3	4 ± 0	12 ± 3	6.9 ± 0.2	2 ± 1
	**171 mM NaCl**				
Non-inoculated	64 ± 5 ^a^	6 ± 1	14 ± 0	9.6 ± 0.4	4 ± 1 ^a^
Hv16	85 ± 6 ^b^	6 ± 1	14 ± 1	9.3 ± 0.2	2 ± 1 ^b^
C58	71 ± 2 ^ab^	6 ± 1	13 ± 1	9.4 ± 0.2	3 ± 0 ^a^
RTE9	91 ± 3 ^b^	5 ± 1	14 ± 2	8.8 ± 0.4	3 ± 1 ^a^

**Table 7:** Length and dry weight of branch and roots, relative water content (RWC), and relative growth rate (RGR) of cuttings of *A. macrostachyum* inoculated with three different bacteria after 60 days. Values correspond to the mean ± S.E. (*n* = 4). Different letters indicate that the mean values are significantly different from each other (Fisher’s LSD test, *p* < 0.05).

	Branch				Root	
Treatment	Length (cm)	Dry Weight (mg)	RWC (%)	RGR (mg g^−1^ day^−1^)	Length (cm)	Dry Weight (mg)
Non-inoculated	82 ± 6	43 ± 3	89 ± 2 ^a^	25 ± 3	44 ± 4 ^b^	8.6 ± 0.6 ^b^
Hv16	88 ± 5	45 ± 3	79 ± 2 ^b^	25 ± 3	52 ± 4 ^a^	10.4 ± 0.6 ^a^
C58	84 ± 6	38 ± 3	87 ± 2 ^a^	21 ± 3	48 ± 5 ^ab^	10.4 ± 0.6 ^a^
RTE9	80 ± 5	39 ± 3	72 ± 2 ^c^	27 ± 2	47 ± 5 ^ab^	8.8 ± 0.7 ^ab^

**Table 8:** Net photosynthetic (A) and stomatal conductance (g_s_) rates, intercellular CO_2_ concentration (C_i_), and intrinsic water use efficiency (_i_WUE) of cuttings of *A. macrostachyum* inoculated with three different bacteria after two months. Values correspond to the mean ± S.E. (*n* = 4). Different letters indicate that the mean values are significantly different from each other (Fisher’s LSD test, *p* < 0.05).

Treatment	A (μmol m^−2^s^−1^)	g_s_ (mmol m^−2^s^−1^)	C_i_ (μmol mol^−1^)	_i_WUE (μmol mol^−1^)
Non-inoculated	6.9 ± 0.2 ^c^	150 ± 20	306 ± 5	45 ± 4 ^c^
Hv16	7.3 ± 0.1 ^bc^	130 ± 20	289 ± 12	61 ± 5 ^ab^
C58	7.9 ± 0.4 ^ab^	150 ± 10	296 ± 6	53 ± 3 ^bc^
RTE9	8.8 ± 0.4 ^a^	123 ± 17	281 ± 11	70 ± 10 ^a^

## Data Availability

The data presented in this study are available in the present article.
